# The Decomposition and Forecasting of Mutual Investment Funds Using Singular Spectrum Analysis

**DOI:** 10.3390/e22010083

**Published:** 2020-01-09

**Authors:** Paulo Canas Rodrigues, Jonatha Pimentel, Patrick Messala, Mohammad Kazemi

**Affiliations:** 1 Department of Statistics, Federal University of Bahia, 40170-110 Salvador, Brazil; jsppimentel9@gmail.com (J.P.); patrickmessala@gmail.com (P.M.); 2 CAST, Faculty of Information Technology and Communication Sciences, Tampere University, FI-33014 Tampere, Finland; 3 Department of Statistics, Faculty of Mathematical Sciences, Shahrood University of Technology, P.O. Box 3619995161 Shahroud, Iran; m.kazemie64@gmail.com

**Keywords:** singular spectrum analysis, robust singular spectrum analysis, time series forecasting, mutual investment funds

## Abstract

Singular spectrum analysis (SSA) is a non-parametric method that breaks down a time series into a set of components that can be interpreted and grouped as trend, periodicity, and noise, emphasizing the separability of the underlying components and separate periodicities that occur at different time scales. The original time series can be recovered by summing all components. However, only the components associated to the signal should be considered for the reconstruction of the noise-free time series and to conduct forecasts. When the time series data has the presence of outliers, SSA and other classic parametric and non-parametric methods might result in misleading conclusions and robust methodologies should be used. In this paper we consider the use of two robust SSA algorithms for model fit and one for model forecasting. The classic SSA model, the robust SSA alternatives, and the autoregressive integrated moving average (ARIMA) model are compared in terms of computational time and accuracy for model fit and model forecast, using a simulation example and time series data from the quotas and returns of six mutual investment funds. When outliers are present in the data, the simulation study shows that the robust SSA algorithms outperform the classical ARIMA and SSA models.

## 1. Introduction

Mutual investment funds provide management services to institutional and individual investors, besides great liquidity for financial investments made in them and low transactional costs [1,2]. These funds can be of fixed or variable income and allow to diversify the assets while reducing unsystematic risk. Fixed income mutual investment funds are of low risk, whereas variable-income mutual investment funds vary in terms of risk but also in terms of returns. In this study, we were interested in analyzing the quotas and returns of six of the largest Brazilian based mutual investment funds—three purely based on stocks: (i) Alaska Black, (ii) APEX Long Biased, and (iii) Brasil Capital; and three balanced funds (usually combining a stock component, a bond component, and sometimes a money market component in a single portfolio): (iv) ADAM Strategy, (v) Gavea Macro, and (vi) SPX Nimitz.

A natural framework for analyzing mutual investment funds, due to its underlying structure, is a time series method.

Singular spectrum analysis (SSA) is a powerful non-parametric technique for time series analysis and forecasting, which incorporates elements of classical time series analysis, multivariate statistics, and matrix algebra. Its main aim is to decompose the original time series into a set of components that can be interpreted as trend components, seasonal components, and noise components [3,4,5,6]. SSA has proven both wide usefulness and applicability across many applications [7,8,9,10,11,12,13,14,15,16,17], being that its scope of application ranges from parameter estimation to time series filtering, synchronization analysis, and forecasting [18].

The SSA methodology for model fit can be summarized in four steps: (i) embedding, which maps the original univariate time series into a trajectory matrix; (ii) singular value decomposition (SVD), which helps decomposing the trajectory matrix into the sum of rank-one matrices; (iii) eigentriple grouping, which helps deciding which of the components are associated to the signal and which are associated to the noise; and (iv) diagonal averaging, which maps the rank-one matrices, associated to the signal, back to time series that can be interpreted as trend, seasonal, or other meaningful components.

SSA results and interpretation, similarly to many other classical time series methods, can be sensitive to data contamination with outliers [19,20]. In those cases, even a small percentage of outliers can make a big difference on the results for model fit and model forecast. Very few attempts have been made in order to access the effect of the presence of outliers in the data while conducting a SSA. One study [21,22] presented some preliminary results on the effect of outliers in singular spectrum analysis, and [23] made a first attempt to robustify the SSA by considering an SVD based on a robust $L_{1}$ norm [24] instead of the $L_{2}$ norm used in the classical algorithm, which they used for model fit.

In this paper we go one step further than [23] and propose a new robust algorithm for SSA that considers the SVD based on the Huber function [25]. Moreover, we propose two robust SSA forecasting algorithms, one based on the the $L_{1}$ norm and another based on the Huber function. Comparisons are made between the classical SSA algorithm, the robust SSA algorithm based on the $L_{1}$ norm (RLSSA), the robust SSA algorithm based on the Huber function (RHSSA), and the classical autoregressive integrated moving average (ARIMA) model, in terms of computational time and accuracy for model fit and model forecast. These comparisons for decomposing and forecasting time series were done by considering a simulation example and the six mutual investment funds mentioned above.

The rest of this paper is organized as follows. Section 2 provides the materials and methods containing the data description, a brief introduction to the ARIMA and SSA methodologies, and the details of the proposed robust SSA algorithm that uses the SVD based on the Huber function. Section 3 presents the results and discussion, wherein the ARIMA, SSA, and robust SSA algorithms are compared in terms of model fit and model forecast, using the six mutual investment funds and the simulation example. The paper closes in Section 4, wherein some conclusions are drawn.

## 2. Materials and Methods

### 2.1. Data

In this paper we consider a dataset that includes daily observations of six mutual investment funds, three based purely on stocks and three balanced funds:

*Stock funds*

- Alaska Black: 3 January 2017–30 August 2019 (*N* = 666 observations).
- APEX Long Biased: 15 April 2013–30 August 2019 (*N* = 1604 observations).
- Brasil Capital: 27 August 2012–30 August 2019 (*N* = 1760 observations).

*Balanced funds*

- ADAM Strategy: 29 April 2016–30 August 2019 (*N* = 838 observations).
- Gavea Macro: 30 June 2008–30 August 2019 (*N* = 2809 observations).
- SPX Nimitz: 01 December 2010–30 August 2019 (*N* = 2199 observations).

The datasets were collected from https://infofundos.com.br/carteira.

### 2.2. ARIMA Model

The autoregressive integrated moving average (ARIMA) models are among the most widely used techniques for time series analysis and forecasting. Such a model depends on three parameters: *p* is the number of lagged observations in the model, i.e., the autoregressive (AR) order; *d* is the number of times that the original observations are differenced, i.e., the integrated (I) degree; and *q* is the size of the moving average window, i.e., the order of the moving average (MA) [26]. This parametric model can then be written as ARIMA(p,d,q), with *p*, *d*, and *q* non-negative integers. Given a time series $Y_{N}=y_{1},\ldots ,y_{N}$, the ARIMA(p,d,q) model can be written as:

(1) $$\left(1-\phi_{1}B^{1}-\cdots -\phi_{p}B^{p}\right){\left(1-B\right)}^{d}y_{t}=c+\left(1+\theta_{1}B^{1}+\cdots +\theta_{q}B^{q}\right)\varepsilon_{t},$$

where $\phi_{1},\ldots ,\phi_{p}$ are the parameters or coefficients of the *p* autoregressive terms; *B* is the time lag operator, or backward shift, which is a linear operator denoted by $B^{k}$ such that $L^{k}y_{t}=y_{t-k}$, t∈Z; $y_{t}$ is the observation at the time point *t*; $c=\mu (1-\phi_{1}-\cdots -\phi_{p})$; μ is the mean of ${\left(1-B\right)}^{d}y_{t}$; $\beta_{1},\ldots ,\beta_{q}$ are the parameters or coefficients of the *q* moving average terms; and $\varepsilon_{t}$ is an error term, usually white noise with variance $\sigma^{2}$.

Alternatively, the model can be written as:

(2) $$\left(1-\phi_{1}B-\cdots -\phi_{p}B^{p}\right){\left(1-B\right)}^{d}\left(y_{t}-\mu t^{d}/d!\right)=\left(1+\theta_{1}B+\cdots +\theta_{q}B^{q}\right)\varepsilon_{t},$$

which is the parametization used in the “arima” function of the software R [27].

### 2.3. Singular Spectrum Analysis

Singular spectrum analysis is a non-parametric technique for model fit and model forecasting that decomposes a time series into a number of components that are summed and interpreted as trend, periodicity, and noise. Similarly to many other time series techniques, SSA can be used for solving a wide range of problems, some of the most relevant being its ability to smooth the original time series, and to separate the signal (i.e., trend and oscillatory components with different amplitudes) from the noise components. Therefore, SSA can be used to analyze and reconstruct smoother noise-free time series that can then be used for model forecasting.

SSA is divided into two interconnected stages: decomposition and reconstruction of the time series. These stages are divided into two sets each, forming a total of four steps: embedding, singular value decomposition (SVD), grouping, and diagonal averaging. The complete algorithm for model fit is described in the following sub-section. Further details can be found in, e.g., [5,6,28].

#### 2.3.1. Decomposition

In the first stage, the (univariate) time series is converted into a high-dimensional matrix called a trajectory matrix, which is then decomposed into the sum of rank-one matrices based on the SVD.

(1) Embedding:

Consider a non-zero time series $Y_{N}=\left\{y_{1},\ldots ,y_{n}\right\}$ with size N>2. Let L(1<L<N) be an integer value called window length and *K* an integer such that the trajectory matrix includes all values; i.e., K=N−L+1. The embedding step is achieved by mapping the original time series into a sequence of *K* vectors with length *L*:

(3) $$Y_{i}={\left(y_{i},\cdots ,y_{i+L-1}\right)}^{T},1\leq i\leq K.$$

Then, the trajectory matrix X, that includes the vectors $Y_{i}$, i=1,…,K, in its columns can be written as:

(4) $$X=\left[Y_{1},\cdots ,Y_{K}\right]={\left(y_{i}j\right)}_{i,j=1}^{LK}=\left[\begin{array}{cccc} y_{1} & y_{2} & \cdots & y_{K}\\ y_{2} & y_{3} & \cdots & y_{K+1}\\ \vdots & \vdots & \ddots & \vdots \\ y_{L} & y_{L+1} & \cdots & y_{N} \end{array}\right].$$

(2) Singular value decomposition: 

Let $S={\mathrm{XX}}^{T}$, $U_{1},\ldots ,U_{L}$ be the eigenvectors of S, and $\lambda_{1}\geq \cdots \geq \lambda_{L}$, its corresponding eigenvalues. If *d* is the number of non-null eigenvalues of S, and considering $V_{i}=X^{T}U_{i}\sqrt{\lambda_{i}}$, we can decompose the trajectory matrix X as:

(5) $$X=\sum_{i=1}^{d}X_{i}=\sum_{i=1}^{d}\sqrt{\lambda_{i}}U_{i}V_{i}^{T}.$$

The decomposition stage can be accomplished either by the eigendecomposition of $X^{T}X$ or by the SVD of X ($X={\mathrm{UDV}}^{T}$, $D=diag(\sqrt{\lambda_{1}},\ldots ,\sqrt{\lambda_{d}})$). A comparison between both decompositions can be found in [29].

#### 2.3.2. Reconstruction

In the second stage, after a separating signal from noise components, a diagonal averaging procedure is conducted in the matrices associated to the signal resulting into the sum of time series components that can then be interpreted as trend or oscillatory components: 

(1) Eigentriple grouping: 

This step consists of identifying the first *r* eigentriples associated with the signal and discarding the d−r eigentriples associated with the noise. Formally, let I=1,…,r and $I^{c}=r+1,\ldots ,d$. The goal of this step is to choose *I* such that the trajectory matrix can be written as:

(6) $$X_{I}=\sum_{i\in I}\sqrt{\lambda_{i}}U_{i}V_{i}^{T}+\epsilon ,$$

where ϵ is the noise term.

The number of eigentriples to conduct the reconstruction is often decided based on w-correlations. We shall say that two series $Y^{\left(1\right)}$ and $Y^{\left(2\right)}$ are approximately separable if all correlations between the rows and the columns of the corresponding trajectory matrices obtained from series $Y^{\left(1\right)}$ and $Y^{\left(2\right)}$ are close to zero. In [5] they considered other characteristics of the quality of separability; namely, the weighted correlation or w-correlation, which is a natural measure of deviation of two series $Y_{T}^{\left(1\right)}$ and $Y_{T}^{\left(2\right)}$ from w-orthogonality:

(7) $$\rho_{12}^{\left(w\right)}=\frac{{\left(Y_{T}^{\left(1\right)},Y_{T}^{\left(2\right)}\right)}_{w}}{∥Y_{T}^{\left(1\right)}{∥}_{w}{∥Y_{T}^{\left(1\right)}∥}_{w}},$$

where $∥Y_{T}^{\left(i\right)}{∥}_{w}=\sqrt{{\left(Y_{T}^{\left(i\right)},Y_{T}^{\left(i\right)}\right)}_{w}}$, i=1,2, and ${\left(Y_{T}^{\left(1\right)},Y_{T}^{\left(2\right)}\right)}_{w}=\sum_{t=1}^{T}w_{t}y_{t}^{\left(1\right)}y_{t}^{\left(2\right)}$ with $w_{t}=\min\left\{t,L,T-t+1\right\}$. If the absolute value of the w-correlation is small, the two series are almost w-orthogonal. If the absolute value of the w-correlation is large, the series are far from being w-orthogonal and are, therefore, badly separable. Further explanation and intuition about this measure can be found in [5,28]. Other proposals for this choice were proposed by, e.g., [30,31]. 

(2) Diagonal averaging: 

In this step, using anti-diagonal averaging on the matrices included in $X_{I}$, the noise-free time series is reconstructed. First, the approximate trajectory matrix $X_{I}$ is transformed into a Hankel matrix. Let $A_{s}=\left\{\left(l,k\right):l+k=s,1\leq l\leq L,1\leq k\leq K\right\}$ and $\#(A_{s})$ be the number of elements in $A_{s}$. The element ${\tilde{x}}_{ij}$ of the new Hankel matrix $\tilde{X}$ is given by:

(8) $${\tilde{x}}_{ij}=\sum_{\left(l,k\right)\in A_{s}}\frac{x_{lk}}{\#(A_{s})}.$$

Next, the Hankel matrix ${\tilde{X}}_{I}$ is transformed into a new series of dimension *N*, and the original time series $Y_{N}$ can be approximated by:

(9) $${\tilde{y}}_{i}=\left\{\begin{array}{cc} {\tilde{x}}_{i1} & \mathrm{for}\;i=1,\ldots ,L,\\ {\tilde{x}}_{Lj} & \mathrm{for}\;i=L+1,\ldots ,N, \end{array}\right.$$

where j=i−L+1.

The reconstructed noise-fee time series can then be used for out-of-sample forecasting.

### 2.4. Robust SSA

Despite knowing that SSA has shown to be superior to traditional model-based methods in many applications, the singular value decomposition (second step of the SSA algorithm) is highly sensitive to data contamination with outliers. Very few studies were made in order to access effects of outliers in SSA and to generalize this methodology [21,22]. A first attempt to robustify the SSA by considering an SVD based on a robust $L_{1}$ norm [24] instead of the $L_{2}$ norm used in the classical algorithm, was proposed by [23]. That robust generalization was compared with the classical SSA algorithm for model fit by these authors. In this subsection we review that robust SSA algorithm proposed by [23] and propose a new robust algorithm for SSA that considers the SVD based on the Huber function [25] and also propose an algorithm for robust SSA model forecasting. While the robust algorithms based on the $L_{1}$ norm are very popular, they have difficulties in handling heavy tail outliers. The robust algorithms based on the Huber function combine the sum of squares loss and the least absolute deviation loss, that is, a quadratic on small errors, but grows linearly for large errors. As a result, the Huber loss function is not only more robust against outliers but also more adaptive for different types of data [32]. Further details and comparisons between the $L_{1}$ and Huber loss functions, among others, can be found in [33]. The R source code is available upon request from the first author of this paper.

#### 2.4.1. Robust SSA Based on the $L_{1}$ Norm

The robust SSA algorithm proposed by [23] replaces the classical SVD based on the least squares $L_{2}$ norm, by the robust SVD algorithm based on the $L_{1}$ norm [24]. This robust SVD is performed iteratively, starting with an initial estimate of the first left singular vector $U_{1}$ and leading to an outlier-resistant approach that also allows for missing data. The robust SVD based on the $L_{1}$ norm is implemented under the function “robustSVD()” from the R package “pcaMethods”.

#### 2.4.2. Robust SSA based on the Huber Function

Here we propose a new alternative to robustify the SSA algorithm, where the least squares SVD in the step two is replaced by the robust SVD based on the Huber function [25]. The Huber loss function [34] can be defined as:

(10) $$L_{\delta }\left(a\right)=\left\{\begin{array}{cc} \frac{1}{2}a^{2} & \mathrm{if}\;|a|\leq \delta \\ \delta \left(\left|a\right|-\frac{1}{2}\delta \right) & \mathrm{if}\;|a|>\delta \end{array},\right.$$

where δ is a parameter that controls the robustness level, and a smaller value of δ usually leads to more robust estimation.

The robust SVD based on the Huber function is a special case of robust regularized SVD and can be obtained with the function “RobRSVD” of the “RobRSVD” R package, in the following way: RobRSVD (data, rough = TRUE, uspar = 0, vspar = 0). In this R implementation, the authors consider δ=1.345, the value commonly used in robust regression that produces 95% efficiency for normal errors [35]. However, numerical studies suggested that the RobRSVD function is not very sensitive to the choice of δ [25]. More details about this robust SVD can be found in [25].

### 2.5. Robust SSA Forecasting Algorithm

The standard recurrent SSA forecasting algorithm assumes that a given observation can be written as a linear combination of the L−1 previous observations [5,6,30]. The coefficients of those linear combinations in the classical SSA forecasting algorithm are obtained based on the left singular vectors, *U*, of the trajectory matrix X. This is valid for SSA because of the orthogonality of the vectors in *U* and of the full rank decomposition of X, which is not the case for the robust SVD algorithms because of their construction and specific properties. To overcome this limitation for the robust SSA algorithms and to be able to obtain out-of-sample forecasts using a robust SSA algorithm, a three stages approach can be conducted:

(i) Use the robust SSA algorithm to obtain a robust approximation for the signal in the trajectory matrix; i.e., conduct the two stages of the robust SSA algorithms, decomposition (using the robust SVD algorithm) and reconstruction, to obtain the noise free (i.e., the signal) trajectory matrix $\tilde{X}$;
(ii) Apply the standard SVD to the matrix $\tilde{X}$ obtained in (i) and obtain $U_{j}^{\nabla }$, the vector of the first L−1 components of $U_{j}$ and $\pi_{j}$, the last component of the vector $U_{j}$, j=1,⋯,r. Then, we can write the coefficient vector $\hat{a}$ as
  (11) $$\hat{a}=\left({\hat{a}}_{L-1},\cdots ,{\hat{a}}_{1}\right){}^{\prime }=\frac{1}{1-\gamma^{2}}\sum_{j=1}^{r}\pi_{j}U_{j}^{\nabla },$$
  where $\gamma^{2}=\sum_{j=1}^{r}\pi_{j}^{2}$.
(iii) The h-steps-ahead out-of-sample recurrent robust SSA forecasts ${\hat{y}}_{N+1},\ldots ,{\hat{y}}_{N+h}$, can be obtained as
  (12) $${\hat{y}}_{t}=\left\{\begin{array}{cc} {\tilde{y}}_{t}, & \mathrm{for}\;t=1,\cdots ,N\\ \sum_{j=1}^{L-1}{\hat{a}}_{j}{\hat{y}}_{t-j}, & \mathrm{for}\;t=N+1,\cdots ,N+h \end{array}\right.$$
  where ${\tilde{y}}_{1},\ldots ,{\tilde{y}}_{N}$, are the fitted values for the reconstructed time series, as obtained from the robust SSA algorithm in (i).

### 2.6. Accuracy Measures

There are several methods and measures for assessing model accuracy based on the behavior of model errors. Here, there are two types of errors:

- Sample errors, called tuning errors;
- Out-of-sample errors, called forecast errors.

Typically, the root mean squared error (RMSE) is used as a criterion for accessing the precision of a model. The RMSE to investigate the quality of the model fit can be written as:

(13) $$RMSE=\sqrt{\frac{1}{N}\sum_{t=1}^{N}{\left(y_{t}-{\tilde{y}}_{t}\right)}^{2}},$$

where $y_{t}$ are the observed values and ${\tilde{y}}_{t}$ the fitted values by the considered model/algorithm (i.e., ARIMA, SSA, robust SSA).

To investigate the forecasting accuracy, let us assume that the last *g* observations are used as a reference (i.e., as test set). Let $N_{0}=N-h-g$. The RMSE to investigate the quality of the forecasting model can be written as:

(14) $$RMSE=\sqrt{\frac{1}{g}\sum_{t=N_{0}+h+1}^{N}{\left(y_{t}-{\tilde{y}}_{t}\right)}^{2}},$$

where $y_{t}$ are the last *g* observed values and ${\tilde{y}}_{t}$ the respective h-steps-ahead forecast values.

## 3. Results and Discussion

In this section, comparisons are made between the classical ARIMA model, the classical SSA algorithm, and the robust SSA algorithms, in terms of computational time and accuracy for model fit and model forecast. These comparisons for decomposing and forecasting time series are done by considering a simulation example and the time series of six mutual investment funds.

Table 1 shows the descriptive statistics for the six mutual investment funds, including the minimum, maximum, and mean returns, being clear that Alaska Black is the fund that shows the largest variation and with the highest mean daily return. On the other end there are Gavea Macro and SPX Nimitz, which show the smallest variations among the considered funds, and low mean returns.

**Figure 1:**
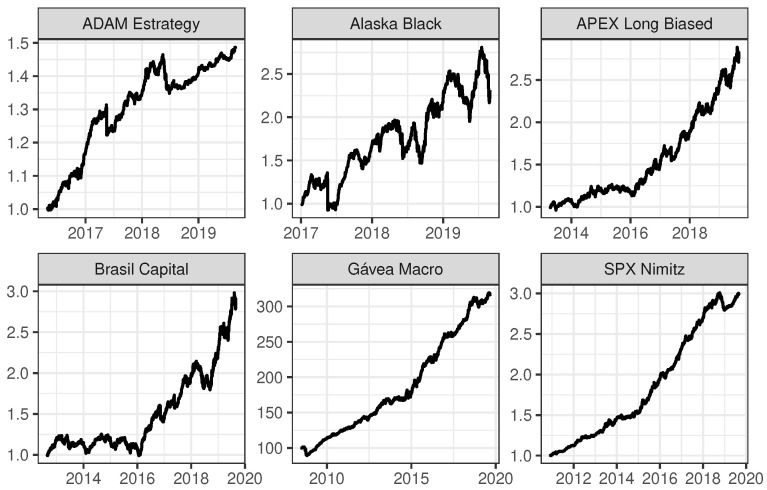
Time series for the returns of the six mutual investment funds, ADAM Strategy, Alaska Black, APEX Long Biased, Brasil Capital, Gávea Macro and SPX Nimitz, from left to right and from top to bottom. The vertical axes show the quota values; i.e., the total net assets of a fund divided by the total number of quotas existing.

In addition to the descriptive measures, Figure 1 shows the behavior of the six investment funds over time. From these plots, it is possible to observe that all funds have an overall growing tendency, with similar patterns for Gavea Macro and SPX Nimitz.

### 3.1. Model Fit

The models/algorithms under comparison for model fit are: (i) ARIMA, (ii) SSA, (iii) robust SSA based on the $L_{1}$ norm (RLSSA), and (iv) robust SSA based on the Huber function (RHSSA).

The parameters of the ARIMA model for each of the six mutual investment funds were estimated with the function “auto.arima” from the R package “forecast” [36].

**Figure 2:**
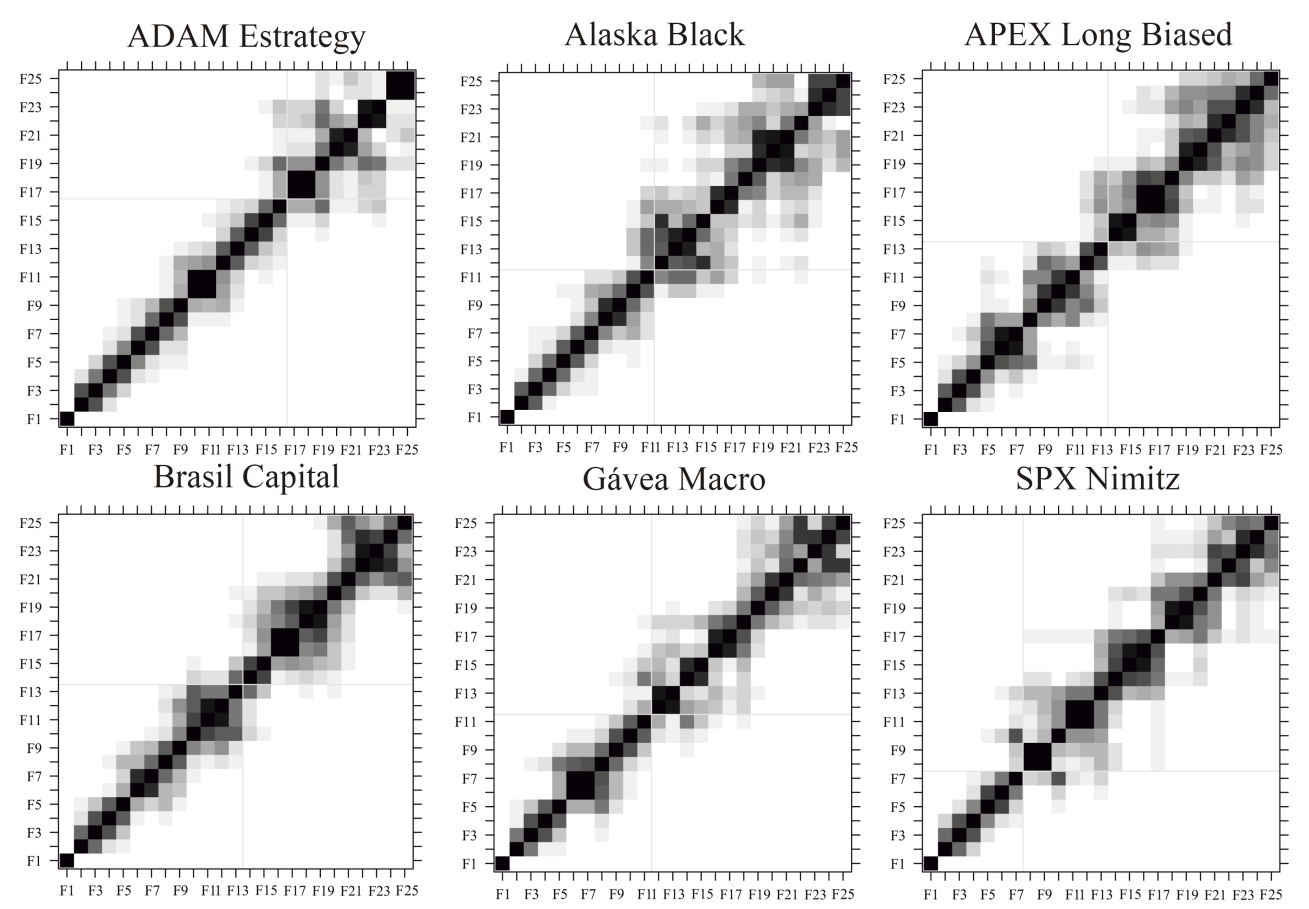
W-correlation matrices for each of the six mutual investment funds, ADAM Strategy, Alaska Black, APEX Long Biased, Brasil Capital, Gávea Macro and SPX Nimitz, from left to right and from top to bottom, considering an window length L=N/20.

**Figure A1:**
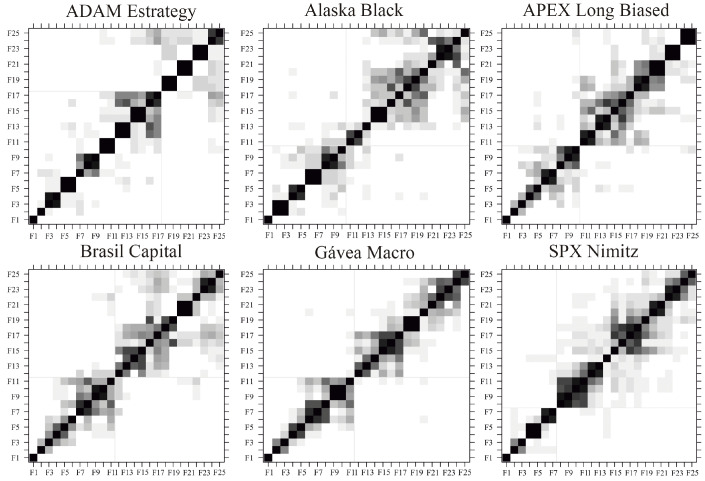
W-correlation matrices for each of the six mutual investment funds, ADAM Strategy, Alaska Black, APEX Long Biased, Brasil Capital, Gávea Macro and SPX Nimitz, from left to right and from top to bottom, considering an window length L=N/2.

For the SSA and robust SSA algorithms, there are two choices to be made by the researcher: (i) the window length *L*; and (ii) the number of eigentriples used for reconstruction *r*. Three values of *L* were chosen for each time series, as defined in Table 2—$L_{1}=N/20$, $L_{2}=N/2$, and $L_{p}$—being the $L_{p}$ obtained from the periodogram, based on the largest cycle for each time series [37] (i.e., about one trimester for ADAM Strategy, one semester for Alaska Black, one year for APEX Long Biased, one quadrimeter for Brasil Capital, one quadrimeter for Gavea Macro, and one quadrimester for SPX Nimitz), and *N* being the time series length. The choice of the number of eigentriples used for reconstruction *r*, for each of the considered window lengths and each of the time series, was done by taking into consideration the the w-correlations among components [5]. Figure 2 shows the w-correlation matrices for each of the six mutual investment funds, considering an window length L=N/20, and Figure A1 of the appendix shows the w-correlation matrices for each of the six mutual investment funds, considering an window length L=N/2. The w-correlation matrices can be obtained with the function “wcor” of the R package “Rssa” [38] and the number of eigentriples *r* should be chosen in order to maximize the separability between signal and noise components; i.e., maximize the w-correlation among signal components, maximize the w-correlation among noise components, and minimize the w-correlation between signal and noise components. A summary of the number of eigentriples used for the reconstruction of each time series for each of the window length considered can be seen in Table 2.

**Figure 3:**
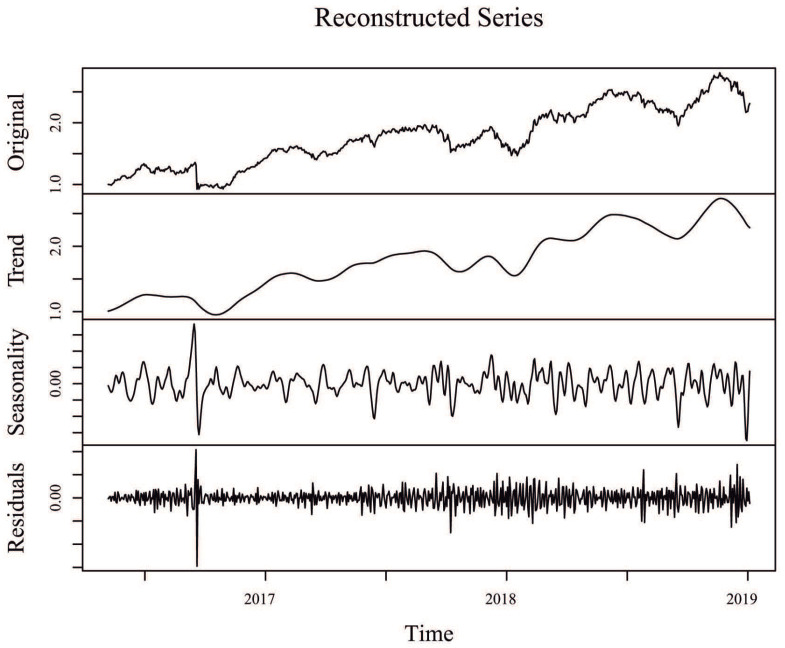
Decomposition of the original time series for the Alaska Black mutual investment fund (top panel), with a trend component (sum of individual trend components, second panel), a seasonal component (sum of individual seasonal components, third panel), and a residual (sum of the remaining components associated to noise, bottom panel), considering an window length L=N/20=33 and r=12 eigentriples for reconstruction.

**Figure A2:**
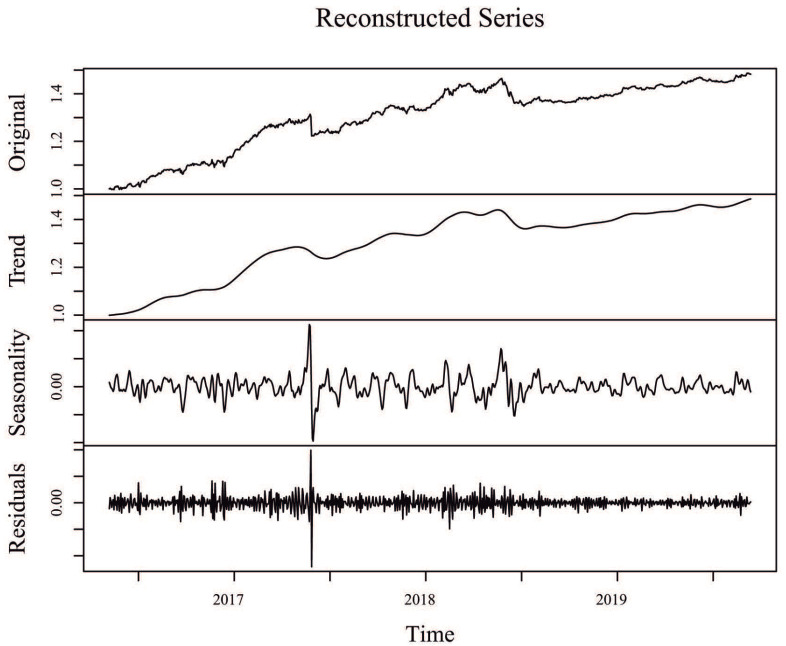
Decomposition of the original time series for the ADAM Strategy mutual investment fund (top panel), with a trend component (sum of individual trend components, second panel), a seasonal component (sum of individual seasonal components, third panel), and a residual (sum of the remaining components associated to noise, bottom panel), considering an window length L=N/20=41 and r=17 eigentriples used for reconstruction.

**Figure A3:**
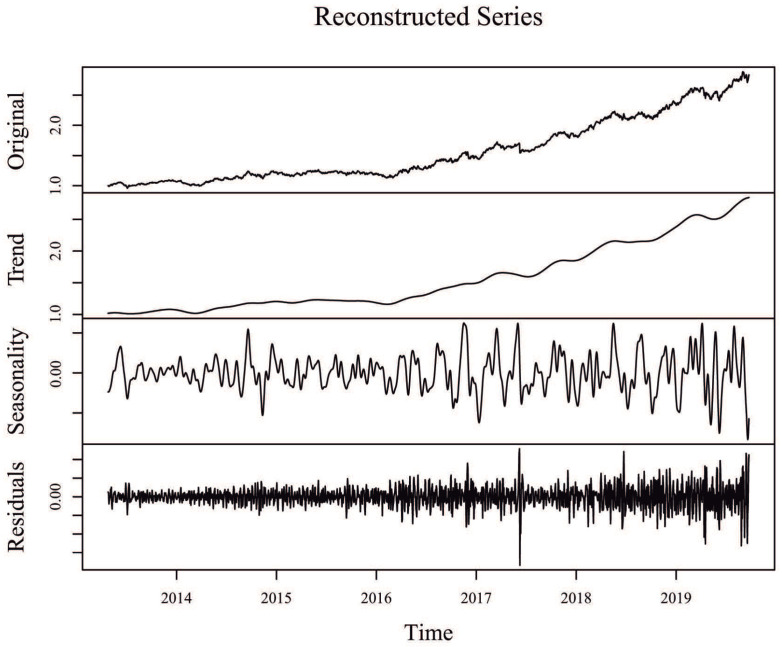
Decomposition of the original time series for the APEX Long Biased mutual investment fund (top panel), with a trend component (sum of individual trend components, second panel), a seasonal component (sum of individual seasonal components, third panel), and a residual (sum of the remaining components associated to noise, bottom panel), considering an window length L=N/20=80 and r=14 eigentriples used for reconstruction.

**Figure A4:**
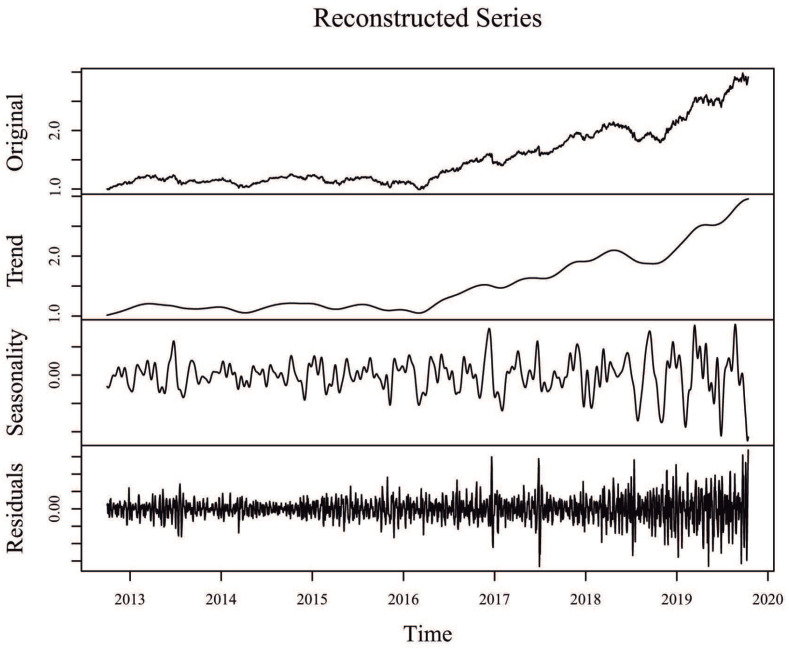
Decomposition of the original time series for the Brasil Capital mutual investment fund (top panel), with a trend component (sum of individual trend components, second panel), a seasonal component (sum of individual seasonal components, third panel), and a residual (sum of the remaining components associated to noise, bottom panel), considering an window length L=N/20=88 and r=12 eigentriples used for reconstruction.

**Figure A5:**
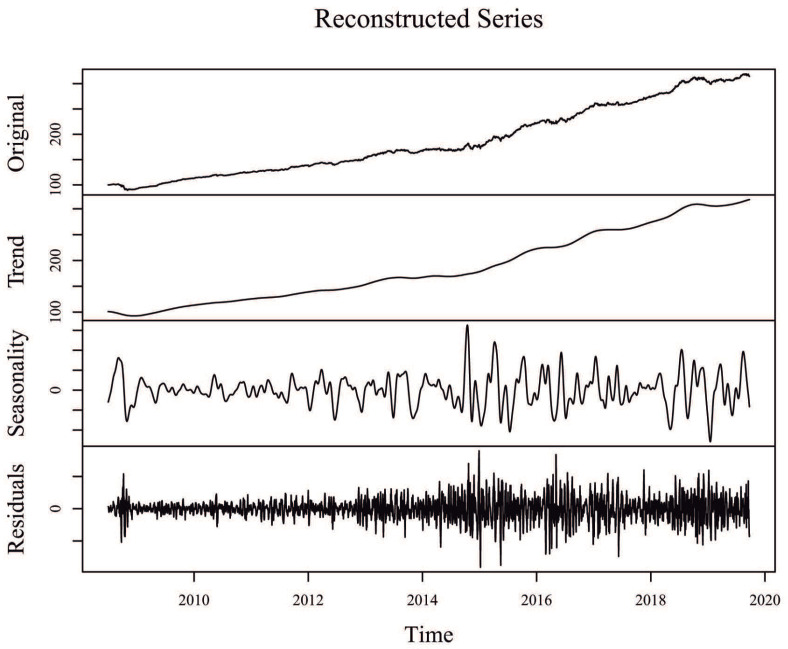
Decomposition of the original time series for the Gavea Macro mutual investment fund (top panel), with a trend component (sum of individual trend components, second panel), a seasonal component (sum of individual seasonal components, third panel), and a residual (sum of the remaining components associated to noise, bottom panel), considering an window length L=N/20=140 and r=12 eigentriples used for reconstruction.

**Figure A6:**
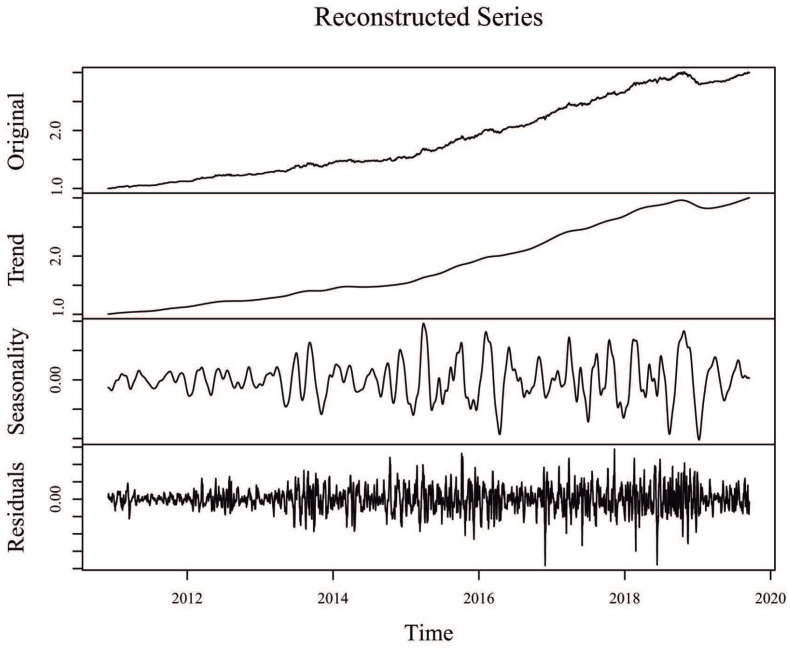
Decomposition of the original time series for the SPX Nimitz mutual investment fund (top panel), with a trend component (sum of individual trend components, second panel), a seasonal component (sum of individual seasonal components, third panel), and a residual (sum of the remaining components associated to noise, bottom panel), considering an window length L=N/20=109 and r=8 eigentriples used for reconstruction.

Since one of the objectives in SSA is to decompose the original time series into interpretable components such as trend and seasonality, plus the noise component that is then discarded, Figure 3 shows the original time series for the Alaska Black mutual investment fund, its trend component (sum of individual trend components), its seasonal component (sum of individual seasonal components), and its residuals (sum of the remaining components associated to noise), considering an window length L=N/20=33 and r=12 eigentriples for reconstruction. Similar SSA decompositions for ADAM Strategy, APEX Long Biased, Brasil Capital, ADAM Strategy, Gavea Macro, and SPX Nimitz—considering the values of window length $L_{1}$ and $r_{1}$ eigentriples used for reconstruction, as defined in Table 2—can be found in Figure A2, Figure A3, Figure A4, Figure A5 and Figure A6 of the appendix, respectively.

In order to evaluate and compare the ability for model fit using the four models, ARIMA, SSA, robust SSA based on the $L_{1}$ norm (RLSSA), and robust SSA based on the Huber function (RHSSA), the root mean square error (RMSE) was calculated for each time series. Table 3 shows the RMSE for model fit by each of the four models applied to each of the six mutual investment funds, considering a window length L=N/2 (Table 2). Table 4 shows the RMSE for model fit by each of the four models applied to each of the six mutual investment funds, considering a window length L=N/20 (Table 2). Table 5 shows the RMSE for model fit by each of the four models applied to each of the six mutual investment funds, considering a window length obtained based on the largest cycle for each time series (Table 2). From the analyzes of these tables, we can conclude that the ARIMA model shows an overall better performance when the window length in the SSA related algorithms is set to be half of the time series (Table 3). However, when the window length is set to be $L_{1}=N/20$ or $L_{p}$ (i.e., equal to the length of the largest cycle), the classical SSA provides the best results, while the ARIMA model and the robust SSA algorithms alternate for the second best performances. For all choices of window length, the two robust SSA algorithms behaved similarly.

Table 6, Table 7 and Table 8 show the computational times for each combination of model/algorithm and mutual investment fund, as presented in Table 3, Table 4 and Table 5, respectively. From the analyzes of these tables, we can conclude that the best performance was obtained by the ARIMA and SSA algorithms. The computational time, for the classic and robust SSA algorithms, increases with the increase of the length *L*. Moreover, for larger trajectory matrices (i.e., considering L=N/2) the robust SSA algorithm based on the Huber function has a lower computational time than the robust SSA algorithm based on the $L_{1}$ norm (Table 6). However, when the trajectory matrices are more rectangular (i.e., considering L=N/20, Table 7, or $L=L_{p}$, Table 8), the robust SSA algorithm based on the $L_{1}$ norm has a much lower computational time (comparable to the ARIMA and SSA computational times) than the robust SSA algorithm based on the Huber function).

**Figure 4:**
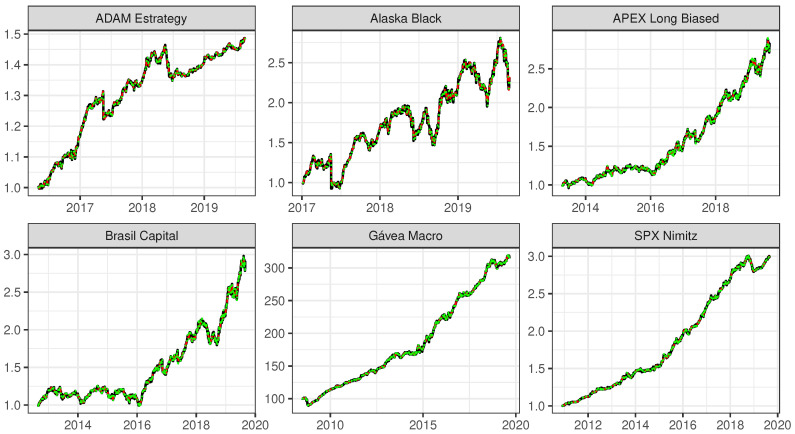
Original time series (black line); smoothed time series after applying the SSA considering L=N/20, with the number of eigentriples *r* as they are defined in Table 2 (red line); and model fit by the ARIMA model (green line), for each of the six mutual investment funds, ADAM Strategy, Alaska Black, APEX Long Biased, Brasil Capital, Gávea Macro and SPX Nimitz, from left to right and from top to bottom. The vertical axes show the quota values.

Figure 4 shows the original time series and the model fit by the SSA model with L=N/20 and by the ARIMA model. We can confirm that both fits are almost overlapped and very near to the original time series, which was expected from the small RMSE showed in Table 4.

### 3.2. Model Forecasting

In this section we compare the forecasting abilities of ARIMA, SSA with L=N/2, SSA with L=N/20, SSA with $L=L_{p}$ based on the largest cycle for each time series, and robust SSA based on the $L_{1}$ norm with L=N/20 and $L_{p}$. The decision for not considering the robust SSA algorithm based on the Huber function was because of its similarity in terms of RMSE with the robust SSA based on the $L_{1}$ norm (Table 3, Table 4 and Table 5) and the much higher computational time (Table 6, Table 7 and Table 8). A similar argument was considered for not presenting the results for the robust SSA algorithm based on the $L_{1}$ norm with L=N/2.

Table 9 shows the RRMSE for model forecasting for each of the six mutual investment funds, considering each of the four models, ARIMA, SSA with L=N/2, SSA with L=N/20, SSA with $L=L_{p}$, and robust SSA based on the $L_{1}$ norm (RLSSA) with L=N/20 and $L_{p}$, considering the window length and engentriples used for reconstruction as defined in Table 2. These values were obtained based on the forecasting of the g=12 observations from each time series, obtained for one, five, and ten steps ahead out-of-sample forecast; i.e., one day ahead, one week ahead, and two weeks ahead.

The overall best performance was obtained with the classic SSA algorithm that considers a lower value for the window length, either L=N/20 or $L=L_{p}$, followed closely by ARIMA and the robust SSA algorithm based on the $L_{1}$ norm. The ARIMA model obtained the best performance in three cases for one-step-ahead forecasting, and the robust SSA algorithm based on the $L_{1}$ norm with L=N/20 yielded the best performance in a couple of time series for five-steps-ahead forecasting. As expected, the RMSE shows an overall increase when increasing the number of steps ahead to be forecast. A possible justification for the similarity between the SSA and robust SSA algorithm can be explained by the possible lack of outliers in the data. Table 10 shows the computational time for model forecasting for each of the six mutual investment funds, considering each of the five models shown in Table 9. As expected, after analyzing the computational times for model fit (Table 6, Table 7 and Table 8), the best performance in terms of computational time for model forecasting was obtained by the the ARIMA and SSA (with lower values for the window length) models and the worse by the robust SSA algorithm based on the $L_{1}$ norm.

### 3.3. Simulation Example

**Figure 5:**
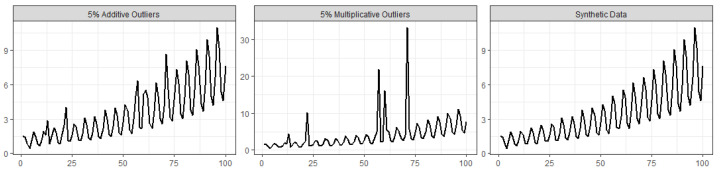
Synthetic data without contamination (**right**), data with 5% additive outliers (**left**), and data with 5% multiplicative outliers (**center**). The vertical axes show the simulated value and the horizontal axes show the index of the simulated observation.

To verify the hypothesis raised in the previous subsection that the similarity between the results from SSA and the robust SSA algorithm can be due to the lack of outliers in the time series, in this subsection we present a simulation example where the methods are compared while analyzing a time series contaminated with outlying observations. The synthetic data were obtained by generating random values from the following function, and then we transformed them into a time series (right-hand plot in Figure 5):

$$\begin{array}{cc} f(t) & =\exp\{0.02t+0.5\sin(2\pi t/5)\}+\epsilon ,\;t=1,\ldots ,100, \end{array}$$

where ϵ is the noise generated from the N(0,0.1). A total of 100 simulated time series were considered.

The data contamination, for illustration purposes, was made by considering additive outliers and magnitude increase outliers in the following way:

- *Additive outliers:* 2%, 5%, and 10% of the time points $y_{i}$ are randomly chosen to be replaced by $2+y_{i}$; i.e., the values of $y_{i}$ are increased by a constant value of 2, resulting in a mild contamination scenario (e.g., (left-hand plot in Figure 5));
- *Magnitude increase:* 2%, 5%, and 10% of the time points $y_{i}$ are randomly chosen to be replaced by $5\times y_{i}$; i.e., the time point magnitude of $y_{i}$ is increased by a factor of 5, resulting in an a quite extreme contamination scenario (e.g., central plot in Figure 5).

Table 11 shows the mean of the root mean square errors for model fit, computed for each of the four models, ARIMA, SSA, robust SSA based on the $L_{1}$ norm, and robust SSA based on the Huber function, for the simulated data, based on 100 runs, using L=24 and r=5, and considering both contamination scenarios with 2, 5, and 10% outliers. As expected, when there is no data contamination, the classic SSA model is the most appropriated. For the mild contamination scenario with additive outliers, the robust SSA algorithms outperform both ARIMA and SSA models, the better performance being more evident when the percentage of the outliers increases. For the more extreme contamination scenario with multiplicative outliers, a similar patters was obtained, the RLSSA being the best robust algorithm, in this simulation example.

Appendix B includes a second simulation scenario where robust SSA algorithm based on the Huber function (RHSSA) outperforms the classic ARIMA and SSA models and the robust SSA algorithm based on the $L_{1}$ norm (RLSSA).

Table 12 shows mean of the root mean square errors for model forecasting (M=1,5,10 steps- ahead), computed for each of ARIMA, SSA, and robust SSA based on the $L_{1}$ norm, for the simulated data, based on 100 runs, using L=24 and r=5. The results for the robust SSA based on the Huber function were not included because of their computational cost and out-performance when compared with the robust SSA based on the $L_{1}$ norm. Again, as expected, the SSA model yielded the best performance for no data contamination. For scenarios with data contamination, the best performance was obtained by the robust SSA forecasting algorithm, with a very large decrease in RMSE in many scenarios.

## 4. Conclusions

In this paper we considered the problem of model fit and model forecasting in time series. In particular, we analyzed six mutual investment funds. Following up on [23], who proposed a robust SSA algorithm by replacing the standard least squares SVD by a robust SVD algorithm based on the $L_{1}$ norm [24] for model fit, we proposed another robust SSA algorithm where the robust SVD based on the Huber function is considered [25]. Moreover, we propose a forecasting strategy for the robust SSA algorithms, based on the linear recurrent SSA forecasting algorithm.

Comparisons were made between the classical SSA algorithm, the robust SSA algorithms, and the classical ARIMA model, both in terms of computational time and accuracy for model fit and model forecast. Those comparisons were made by using daily observations of six mutual investment funds, and a synthetic data set where the time series were contaminated with outlying observations.

For model fit of the six mutual investment funds, the best results were obtained for the SSA model when the window length *L* was set to be equal to the length of the time series divided by 20, or when the window length is defined as the length of the largest cycle in the time series. The ARIMA model and the robust SSA algorithms alternated for the second best performance. For model forecasting of the six mutual investment funds, the best overall performance was obtained for the classic SSA model considering a lower value for the window length, L=N/20 or $L_{p}$, followed closely by the ARIMA model and the robust SSA algorithm based on the $L_{1}$ norm.

Based on the similarity between the results from the classic SSA model and the robust SSA algorithms, both for model fit and model forecasting, one may assume that the time series data from the six mutual investment funds had no or little data contamination. To access that hypothesis and to better illustrate the usefulness of the robust SSA algorithms, using a scenario with known and controlled outliers, a simulation study and its results were presented in this article. For both mild and and more extreme contamination scenarios, the robust SSA algorithms clearly outperformed the classical AMMI and SSA models, both for model fit and for model forecasting. Another important advantage of the robust SSA algorithms, because of their use of the robust SVD, is that they allow for missing values.

In terms of computational time, the SSA model gives the best performance, the robust algorithms being the most time consuming. A possible future development to reduce the computational time in the robust SSA algorithms is to consider a similar strategy as in [39], where a randomized SVD algorithm was used to speed up the SSA algorithm.

The usefulness of the proposed approach, regarding the forecasting case, can be assessed based on forecasting competitions (e.g., [40]) or large scale forecasting studies (see, e.g., [41]).

The methodology and results presented in this paper are of great generality and can be applied to other time series applications.

## Figures and Tables

**Table 1:** Descriptive measures for returns of the six mutual investment funds.

Investment Fund	Minimum	Mean	Maximum	Standard deviation
ADAM Strategy	−6.26%	0.05%	1.63%	0.0045%
Alaska Black	−29.62%	0.16%	9.80%	0.0240%
APEX Long Biased	−8.60%	0.07%	3.72%	0.0085%
Brasil Capital	−7.55%	0.07%	3.42%	0.0094%
Gavea Macro	−2.22%	0.04%	2.36%	0.0033%
SPX Nimitz	−1.92%	0.05%	1.42%	0.0030%

**Table 2:** Window length $L_{1}$, $L_{2}$, and $L_{p}$, and number of eigentriples *r* considered for model fit and model forecast for each of the mutual investment funds.

Investment Fund	*n*	$L_{1}$	$r_{1}$	$L_{2}$	$r_{2}$	$L_{p}$	$r_{p}$
ADAM Strategy	838	41	17	419	18	60	13
Alaska Black	666	33	12	333	11	125	8
APEX Long Biased	1604	80	14	802	11	250	11
Brasil Capital	1760	88	12	880	12	80	13
Gavea Macro	2809	140	12	1404	12	80	12
SPX Nimitz	2199	109	8	1099	8	80	11

**Table 3:** Root mean square error for each of the six mutual investment funds, considering each of the four models, ARIMA, SSA, robust SSA based on the $L_{1}$ norm (RLSSA), and robust SSA based on the Huber function (RHSSA), for the window length $L_{2}=N/2$ and considering $r_{2}$ engentriples for reconstruction as defined in Table 2.

Investment Fund	ARIMA	SSA	RLSSA	RHSSA
ADAM Strategy	0.0057	0.0075	0.0088	0.0076
Alaska Black	0.0402	0.0450	0.0508	0.0476
APEX Long Biased	0.0160	0.0294	0.0318	0.0320
Brasil Capital	0.0170	0.0338	0.0429	0.0346
Gavea Macro	0.6756	1.9758	2.1486	2.0016
SPX Nimitz	0.0063	0.0197	0.0239	0.0207

**Table 4:** Root mean square error for each of the six mutual investment funds, considering each of the four models, ARIMA, SSA, robust SSA based on the $L_{1}$ norm (RLSSA), and robust SSA based on the Huber function (RHSSA), for the window length $L_{1}=N/20$ and considering $r_{1}$ engentriples for reconstruction as defined in Table 2.

Investment Fund	ARIMA	SSA	RLSSA	RHSSA
ADAM Strategy	0.0057	0.0024	0.0034	0.0034
Alaska Black	0.0402	0.0190	0.0244	0.0234
APEX Long Biased	0.0160	0.0107	0.0124	0.0116
Brasil Capital	0.0170	0.0124	0.0143	0.0133
Gavea Macro	0.6756	0.6508	0.7716	0.7432
SPX Nimitz	0.0063	0.0066	0.0078	0.0077

**Table 5:** Root mean square error for each of the six mutual investment funds, considering each of the four models, ARIMA, SSA, robust SSA based on the $L_{1}$ norm (RLSSA), and robust SSA based on the Huber function (RHSSA), for the window length $L_{p}$ (i.e., the length of the largest cycle) and considering $r_{p}$ engentriples for reconstruction as defined in Table 2.

Investment Fund	ARIMA	SSA	RLSSA	RHSSA
ADAM Strategy	0.0057	0.0038	0.0046	0.0045
Alaska Black	0.0402	0.0415	0.0482	0.0459
APEX Long Biased	0.0160	0.0185	0.0196	0.0190
Brasil Capital	0.0170	0.0123	0.0139	0.0132
Gavea Macro	0.6756	0.5049	0.5997	0.5986
SPX Nimitz	0.0063	0.0049	0.0058	0.0057

**Table 6:** Computational time, in minutes, for each of the six mutual investment funds, considering each of the four models, ARIMA, SSA, robust SSA based on the $L_{1}$ norm (RLSSA), and robust SSA based on the Huber function (RHSSA), for the window length $L_{2}=N/2$ and considering $r_{2}$ engentriples for reconstruction as defined in Table 2.

Investment Fund	ARIMA	SSA	RLSSA	RHSSA
ADAM Strategy	0.0010	0.0052	15.563	14.232
Alaska Black	0.0018	0.0042	7.5859	6.8834
APEX Long Biased	0.0175	0.0320	195.27	61.031
Brasil Capital	0.0226	0.0366	287.80	83.821
Gavea Macro	0.0057	0.1584	1605.2	632.84
SPX Nimitz	0.0022	0.0618	616.75	120.83

**Table 7:** Computational time, in minutes, for each of the six mutual investment funds, considering each of the four models, ARIMA, SSA, robust SSA based on the $L_{1}$ norm (RLSSA), and robust SSA based on the Huber function (RHSSA), for the window length $L_{1}=N/20$ and considering $r_{1}$ engentriples for reconstruction as defined in Table 2.

Investment Fund	ARIMA	SSA	RLSSA	RHSSA
ADAM Strategy	0.0010	0.0025	0.1257	68.384
Alaska Black	0.0018	0.0031	0.0669	16.794
APEX Long Biased	0.0175	0.0039	1.2952	530.43
Brasil Capital	0.0226	0.0048	1.9145	629.79
Gavea Macro	0.0057	0.0088	10.823	1441.1
SPX Nimitz	0.0022	0.0050	3.7450	375.29

**Table 8:** Computational time, in minutes, for each of the six mutual investment funds, considering each of the four models, ARIMA, SSA, robust SSA based on the $L_{1}$ norm (RLSSA), and robust SSA based on the Huber function (RHSSA), for the window length $L_{p}$ (i.e., the length of the longest cycle) and considering $r_{p}$ engentriples for reconstruction as defined in Table 2.

Investment Fund	ARIMA	SSA	RLSSA	RHSSA
ADAM Strategy	0.0010	0.0024	0.3371	65.149
Alaska Black	0.0018	0.0026	1.6994	3.3270
APEX Long Biased	0.0175	0.0078	26.826	115.14
Brasil Capital	0.0226	0.0099	2.0020	804.16
Gavea Macro	0.0057	0.0126	3.4485	1718.4
SPX Nimitz	0.0022	0.0078	3.4937	905.16

**Table 9:** Root mean square error for model forecasting for each of the six mutual investment funds, considering the models ARIMA, SSA with L=N/2, SSA with L=N/20, SSA with $L_{p},$ robust SSA based on the $L_{1}$ norm (RLSSA) with L=N/20, and RSSA with $L_{p}$, and their respective engentriples, as defined in Table 2.

Investment Fund	ARIMA	SSA $\frac{N}{2}$	SSA $\frac{N}{20}$	SSA $L_{p}$	RLSSA $\frac{N}{20}$	RLSSA $L_{p}$
	one-step-ahead					
ADAM Strategy	0.0027	0.0036	0.0029	0.0047	0.0048	0.0048
Alaska Black	0.0712	0.2118	0.0638	0.1357	0.1138	0.178
APEX Long Biased	0.0426	0.1778	0.0544	0.0646	0.0663	0.0576
Brasil Capital	0.0436	0.0496	0.0590	0.0573	0.0545	0.0512
Gavea Macro	1.1670	2.3104	1.5536	1.2571	1.1532	1.6582
SPX Nimitz	0.0081	0.0278	0.0061	0.0061	0.0061	0.0074
	five-step-ahead					
ADAM Strategy	0.0056	0.0047	0.0058	0.0038	0.0089	0.0057
Alaska Black	0.2031	0.2990	0.1800	0.1848	0.2120	0.2365
APEX Long Biased	0.1184	0.1965	0.0578	0.0724	0.0830	0.0577
Brasil Capital	0.1277	0.0481	0.0704	0.0669	0.0693	0.0615
Gavea Macro	2.4007	2.8585	2.0509	1.8165	1.2367	2.3534
SPX Nimitz	0.0275	0.0292	0.0075	0.0077	0.0076	0.0108
	ten-step-ahead					
ADAM Strategy	0.0057	0.0087	0.0055	0.0086	0.0111	0.0091
Alaska Black	0.2958	0.3795	0.2201	0.0263	0.3311	0.3329
APEX Long Biased	0.2012	0.2162	0.0929	0.0706	0.1020	0.0555
Brasil Capital	0.1998	0.0460	0.1100	0.1101	0.0844	0.0700
Gavea Macro	3.2948	3.6784	2.6578	2.7515	2.8015	2.5541
SPX Nimitz	0.0467	0.0314	0.0166	0.0120	0.0103	0.0170

**Table 10:** Computational time, in minutes, for the model for each of the six mutual investment funds, considering the models ARIMA, SSA with L=N/2, SSA with L=N/20, SSA with $L_{p},$ robust SSA based on the $L_{1}$ norm (RLSSA) with L=N/20, and RSSA with $L_{p}$, and their respective engentriples, as defined in Table 2.

Investment Fund	ARIMA	SSA $\frac{N}{2}$	SSA $\frac{N}{20}$	SSA $L_{p}$	RLSSA $\frac{N}{20}$	RLSSA $L_{p}$
	one-step-ahead					
ADAM Strategy	0.0123	0.1231	0.0277	0.0253	39.768	58.804
Alaska Black	0.0222	0.0549	0.0183	0.0267	30.516	45.948
APEX Long Biased	0.2106	0.4888	0.0613	0.1752	176.18	692.18
Brasil Capital	0.2712	0.8409	0.0644	0.0648	212.60	295.20
Gavea Macro	0.0681	2.7338	0.1687	0.0976	698.34	857.58
SPX Nimitz	0.0265	1.2750	0.0774	0.0740	420.23	584.59
	five-step-ahead					
ADAM Strategy	0.0129	0.0879	0.0222	0.0256	44.019	56.524
Alaska Black	0.0181	0.0531	0.0150	0.0246	32.351	58.674
APEX Long Biased	0.2203	0.4909	0.0682	0.1840	250.85	675.41
Brasil Capital	0.2620	0.6400	0.0764	0.0675	314.59	290.72
Gavea Macro	0.0702	2.7839	0.1460	0.1034	988.02	858.96
SPX Nimitz	0.0348	1.3029	0.0805	0.0755	537.94	572.93
	ten-step-ahead					
ADAM Strategy	0.0089	0.0924	0.0344	0.0261	45.729	46.518
Alaska Black	0.0156	0.0469	0.0184	0.0263	28.140	54.289
APEX Long Biased	0.1775	0.5057	0.0678	0.1906	198.27	638.13
Brasil Capital	0.2103	0.6628	0.0726	0.0679	244.06	307.30
Gavea Macro	0.0532	2.6942	0.1724	0.1060	761.49	520.66
SPX Nimitz	0.0243	1.2388	0.0634	0.0786	407.61	316.60

**Table 11:** Mean of the root mean square errors for model fit, computed for each of the four models, ARIMA, SSA, robust SSA based on the $L_{1}$ norm, and robust SSA based on the Huber function, for the simulated data, based on 100 runs, using L=24 and r=5.

% of Data Contamination	Shift	ARIMA	SSA	RLSSA	RHSSA
0%	-	0.715	0.083	0.109	0.127
2%	$y_{i}+2$	0.612	0.149	0.119	0.133
5%	$y_{i}+2$	0.640	0.236	0.134	0.148
10%	$y_{i}+2$	0.675	0.364	0.179	0.232
2%	$y_{i}\times 5$	1.206	1.235	0.126	0.389
5%	$y_{i}\times 5$	1.828	2.289	0.167	0.929
10%	$y_{i}\times 5$	2.384	3.404	0.425	1.463

**Table 12:** Mean of the root mean square errors for model forecasting (M=1,5, and 10 steps-ahead), computed for each of the four models, ARIMA, SSA, robust SSA based on the $L_{1}$ norm, and robust SSA based on the Huber function, for the simulated data, based on 100 runs, using L=24 and r=5.

M	% of Cont.	Shift	Method		
			ARIMA	SSA	RLSSA
M = 1	0%	-	1.685	0.125	0.245
	5%	$y_{i}+2$	0.843	0.475	0.330
	10%	$y_{i}+2$	0.793	0.596	0.426
	5%	$y_{i}\times 5$	3.960	8.461	0.358
	10%	$y_{i}\times 5$	4.359	9.692	0.652
M = 5	0%	-	1.631	0.122	0.222
	5%	$y_{i}+2$	0.984	0.475	0.307
	10%	$y_{i}+2$	0.768	0.586	0.413
	5%	$y_{i}\times 5$	3.789	538.447	0.323
	10%	$y_{i}\times 5$	3.853	17.670	0.720
M = 10	0%	-	1.381	0.127	0.244
	5%	$y_{i}+2$	1.320	0.601	0.358
	10%	$y_{i}+2$	1.148	0.698	0.474
	5%	$y_{i}\times 5$	3.486	22.695 *	4.015
	10%	$y_{i}\times 5$	3.694	622.783	2.320

* 10% trimed mean. The mean value is $1.566\times 10^{6}$.

**Figure A7:**
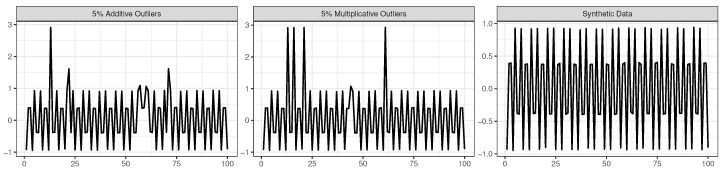
Synthetic data without contamination (**right**), data with 5% additive outliers (**left**), and data with 5% multiplicative outliers (**center**). The vertical axes show the simulated value and the horizontal axes show the index of the simulated observation.

## Abbreviations

The following abbreviations are used in this manuscript:

ARIMA	autoregressive integrated moving average
SSA	singular spectrum analysis
SVD	singular value decomposition
RHSSA	robust SSA algorithm based on the Huber function
RLSSA	robust SSA algorithm based on the $L_{1}$ norm
RMSE	root mean squared error

## Appendix B

A second synthetic dataset was obtained by generating random values from the following function and then transforming them into a time series:

$$\begin{array}{c} f\left(t\right)=cos\left(2\pi wt+\phi \right)+\epsilon ,\;t=1,\ldots ,100, \end{array}$$

with w=3/8, ϕ=π/8 and ϵ the noise generated from the N(0,0.1) (right-hand side of Figure A7). A total of 100 simulated time series were considered.

The data contamination was done in the same manner as described before. An example of 5% additive outliers scenario can be found on the left-hand plot of Figure A7, and an example of 5% multiplicative outliers scenario can be found on the central plot of Figure A7. The results for the root mean square errors for model fit, computed for each of the four models, ARIMA, SSA, robust SSA based on the $L_{1}$ norm, and robust SSA based on the Huber function, can be found in Table A1.

**Table A1:** Mean of the root mean square errors for model fit, computed for each of the four models, ARIMA, SSA, robust SSA based on the $L_{1}$ norm, and robust SSA based on the Huber function, for the simulated data, based on 100 runs, using L=24 and r=2.

% of Data Contamination	Shift	ARIMA	SSA	RLSSA	RHSSA
0%	-	0.1045	0.0097	0.0099	0.0104
2%	$y_{i}+2$	0.277	0.071	0.058	0.019
5%	$y_{i}+2$	0.351	0.113	0.096	0.032
10%	$y_{i}+2$	0.465	0.161	0.197	0.055
2%	$y_{i}\times 5$	0.279	0.108	0.026	0.018
5%	$y_{i}\times 5$	0.386	0.193	0.052	0.040
10%	$y_{i}\times 5$	0.484	0.338	0.075	0.098
